# Testing the Growth Rate Hypothesis in Vascular Plants with Above- and Below-Ground Biomass

**DOI:** 10.1371/journal.pone.0032162

**Published:** 2012-03-13

**Authors:** Qiang Yu, Honghui Wu, Nianpeng He, Xiaotao Lü, Zhiping Wang, James J. Elser, Jianguo Wu, Xingguo Han

**Affiliations:** 1 State Key Laboratory of Forest and Soil Ecology, Institute of Applied Ecology, Chinese Academy of Sciences, Shenyang, China; 2 State Key Laboratory of Vegetation and Environmental Change, Institute of Botany, Chinese Academy of Sciences, Beijing, China; 3 Institute of Geographic Sciences and Natural Resources Research, Chinese Academy of Sciences, Beijing, China; 4 School of Life Sciences, Arizona State University, Tempe, Arizona, United States of America; 5 Sino-US Center for Conservation, Energy and Sustainability Science (SUCCESS), Inner Mongolia University, Hohhot, Inner Mongolia, China; University College London, United Kingdom

## Abstract

The growth rate hypothesis (GRH) proposes that higher growth rate (the rate of change in biomass per unit biomass, μ) is associated with higher P concentration and lower C∶P and N∶P ratios. However, the applicability of the GRH to vascular plants is not well-studied and few studies have been done on belowground biomass. Here we showed that, for aboveground, belowground and total biomass of three study species, μ was positively correlated with N∶C under N limitation and positively correlated with P∶C under P limitation. However, the N∶P ratio was a unimodal function of μ, increasing for small values of μ, reaching a maximum, and then decreasing. The range of variations in μ was positively correlated with variation in C∶N∶P stoichiometry. Furthermore, μ and C∶N∶P ranges for aboveground biomass were negatively correlated with those for belowground. Our results confirm the well-known association of growth rate with tissue concentration of the limiting nutrient and provide empirical support for recent theoretical formulations.

## Introduction

Carbon (C), nitrogen (N) and phosphorus (P) are very important elements for living organisms [1]. Their relative use in biomass (i.e. their C∶N∶P stoichiometry) reflects a complex interplay of evolutionary processes [2] coupled to phenotypic plasticity that is driven by patterns of element supply from the environment or diet. Thus, it is increasingly recognized that the values and ranges of C∶N∶P ratios in an organism are important determinants of the ecological niche. Indeed, C∶N∶P stoichiometry, and especially N∶P ratio, is a powerful factor underlying diverse ecological processes [3], such as population stability [4], competitive interactions [5], community organization [6], trophic dynamics [7], litter decomposition [8], [9], nutrient limitation [10], [11], and biogeochemical cycling [12]. Thus, it is important to understand the underlying biological factors that drive observed variation in C∶N∶P ratios in organisms.

Considerable recent work has proposed specific connections between C∶N∶P stoichiometry and growth rate [1]. Growth rate is a central integrating parameter of overall life history strategy [13] and is closely linked to fitness [14]. Initiated from the study of crustacean zooplankton, the growth rate hypothesis (GRH) proposes that fast-growing organisms have low biomass C∶P and N∶P ratios [1], [3] because of differential allocaiton to P-rich ribosomal RNA. By integrating ecological consequences with cellular and genetic mechanisms, the GRH broadened the use of stoichiometric concept in evolutionary studies [3], [15], [16], providing a unifying thread connecting genes to ecosystems. The GRH has been intensively tested and generally supported via both theoretical and empirical analysis in zooplankton, arthropods, and bacteria [3], [16]–[20]. However, the applicability of the hypothesis to photoautotrophs is not entirely clear, especially given the fact that storage materials in plants may obscure the associations between C∶N∶P stoichiometry and growth rate [1], [21], [22]. So, it is not clear whether the relationships between growth rate and C∶N∶P observed in the world of bacteria and zooplankton would also be observed for plants.

Diverse comprehensive reviews have shown that foliar N content in vascular plants tends to increase less than proportionately with P content [23]–[27]; thus, nutrient-rich foliage tends to have low N∶P ratio, suggesting that the GRH has validity in the realm of vascular plants. However, not all studies in plants provide consistent support for the GRH. For example, Matzek and Vitousek's data for pine species showed that it was plant protein∶RNA ratio but not foliar N∶P ratio that was significantly correlated (negatively) with growth rate [28]. Thus, the interactions between N∶P stoichiometry and growth rate require further study.

Ågren proposed to adapt the GRH to plants via a quantitative model of relationship between growth rate (μ) and N∶C (*R*_N∶C_), P∶C (*R*_P∶C_), N∶P (*R*_N∶P_) with the following four equations [21]:
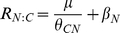
(1)

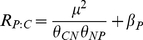
(2)

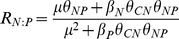
(3)

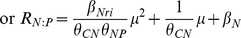
(4)where *θ_CN_* represents the rate of C assimilated by proteins; *θ_NP_* represents the rate of proteins assimilated by ribosomes, please see more details for these equations in [21]. Equation 1 predicts that N∶C ratio is a linear increasing function of μ. Equation 2 predicts that P∶C changes quadratically with μ. Thus, the N∶P ratio is predicted to be a unimodal function of μ, increasing for small values of μ, reaching a maximum, and then decreasing.

Previous studies have provided considerable evidence for positive relationships between N∶C or P∶C with growth rate of plants [21]. However, the relationship between N∶P and growth rate is unclear. Only a few experiments have tested the GRH in vascular plants [21], [22], [28], [29], especially under both N- and P- limited conditions [21], [22]. Unfortunately, even among those limited studies, the results are mixed. N∶P ratio of birch seedlings decreased with μ when P was limiting but increased with μ when N was limiting [21], suggesting the relationship between N∶P and μ varies considerably under different nutrient conditions. Consistent with Ågren's theory, Cernusak *et al.* [29] found that seedlings of 13 tropical tree and liana species showed hump-shaped relationships between N∶P ratio and the relative growth rate. However, Matzek and Vitousek [28] found no relationship between μ and N∶P in greenhouse experiments across 14 species. Furthermore, most studies have focused only on foliage and above-ground biomass. To our knowledege, no study has been done to test the relationship between root C∶N∶P stoichiometry and μ. Thus, not only the GRH but also Ågren's model need more comprehensive testing in terestrial vascular plants, especially for belowground tissues.

To test the GRH and Ågren's theory in vascular plants, here we conducted a sand culture experiment in the temperate steppe of Inner Mongolia. Three grassland plants were planted in sand pots with various N and P levels to examine the relationship between C∶N∶P and growth rates of aboveground, belowground and total biomass under the variation of N and P.

## Results

### Comparison of μ along N and P enrichment levels across the three species

**Figure 1:**
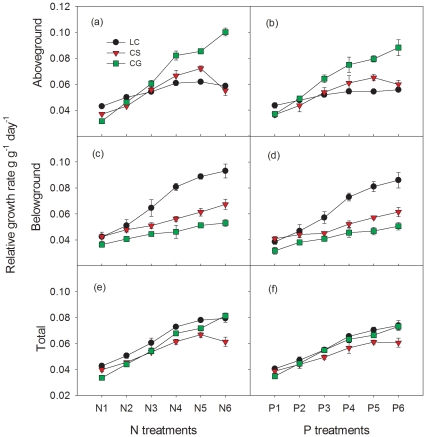
The comparison of relative growth rate of aboveground (a, b), belowground (c, d) and total biomass (e, f) along N and P enrichment levels across the three species. The three species are: *Leymus chinensis* (LC), *Cleistogenes squarrosa* (CS) and *Chenopodium glaucum* (CG).

Relative growth rate of each species increased significantly with increasing N and P availability for aboveground, belowground, and total biomass across low N and P fertilization levels (Fig. 1). However, for aboveground and total biomass at high levels of N and P, relative growth rate did not increase significantly or decreased with increasing N and P fertilization for *Leymus chinensis* and *Cleistogenes squarrosa*. The existence of N or P limitation was estimated by the changes of total biomass in response to N or P fertilization. If total biomass under a certain N or P treatment did not increase significantly compared to the lower N or P treatment, the treatment was delineated as an “excess treatment” and all the lower treatments were delineated as N or P limiting treatments. For *Leymus chinensis*, N5, N6, P5 and P6 were excess N and P levels respectively, i.e. N0–N4 were N limiting treatments while P0–P4 were P limiting treatments; for *Cleistogenes squarrosa*, N6 and P6 were excess N and P levels, i.e. N0–N5 were N limiting treatments while P0–P5 were P limiting treatments. For *Chenopodium glaucum* μ of aboveground, belowground, and total biomass all increased with increasing N and P availability across all the fertilization levels, indicating that all N treatments were N limiting and all P treatments were P limiting for *Chenopodium glaucum*.

### Relationships between μ and N∶C of the three species

**Figure 2:**
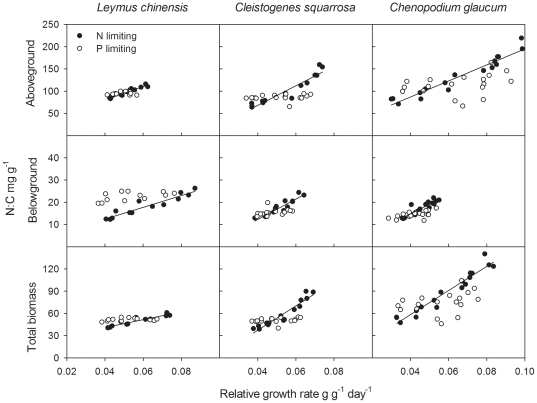
Relationships between relative growth rate and N∶C for aboveground, belowground and total biomass. See analysis results in Table 1.

Consistent with the predictions of equation 1, aboveground, belowground and total biomass N∶C ratio increased linearly as a function of μ for each of the three species when N was limiting (Fig. 2 and Table 1). However, when excess N treatments were included, no significant relationships were found for *Leymus chinensis* and *Cleistogenes squarrosa*.

**Table 1:** Results of regression analyses of the relationships relative growth rate and C∶N∶P of three species in the sand culture experiment.

Plant parts	Experiment	Species	N∶C		P∶C	
			*P*	*R* ^2^	*P*	*R* ^2^
aboveground	N limiting	LC	<0.0001	0.93	n.s.	0.1
		CS	<0.0001	0.90	<0.0001	0.84
		CG	<0.0001	0.94	<0.0001	0.88
	P limiting	LC	n.s.	0.02	= 0.0001	0.87
		CS	n.s.	0.02	<0.0001	0.82
		CG	n.s.	0.10	<0.0001	0.78
belowground	N limiting	LC	<0.0001	0.94	<0.0001	0.91
		CS	<0.0001	0.90	= 0.0059	0.50
		CG	<0.0001	0.82	= 0.0232	0.39
	Plimiting	LC	= 0.0013	0.49	<0.0001	0.95
		CS	n.s.	0.18	<0.0001	0.90
		CG	= 0.0113	0.34	<0.0001	0.84
Total	N limiting	LC	<0.0001	0.88	<0.0001	0.88
		CS	<0.0001	0.91	= 0.0091	0.49
		CG	<0.0001	0.94	<0.0001	0.83
	P limiting	LC	n.s.	0.09	<0.0001	0.93
		CS	n.s.	0.15	<0.0001	0.95
		CG	n.s.	0.13	<0.0001	0.88

For each of the three species, analysis was conducted without the data in excess N or P treatments. No significant difference (n.s.) represents *P*>0.05. *Leymus chinensis* (LC), *Cleistogenes squarrosa* (CS) and *Chenopodium glaucum* (CG).

No significant relationships were found between aboveground and total biomass N∶C and μ for any of the three species when P was limiting (Fig. 2 and Table 1). For belowground biomass of *Cleistogenes squarrosa*, no significant correlation between N∶C and μ was found in the P treatments (Table 1); however, significant relationships were found for the other two species.

### Relationships between μ and P∶C of the three species

**Figure 3:**
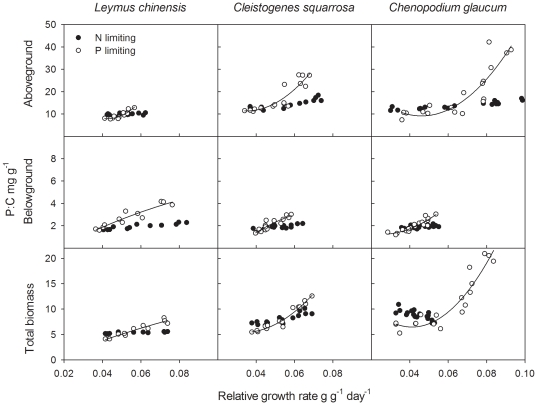
Relationships between relative growth rate and P∶C for aboveground, belowground and total biomass. See analysis results in Table 1.

All aboveground, belowground, and total biomass P∶C ratios increased as a nonlinear function of μ either when N or P was limiting (with the only exception being *Leymus chinensis* aboveground biomass in the N treatments) (Fig. 3 and Table 1). These results are qualitatively consistent with the predictions of equation 2, with a lower Akaike information criterion (AIC) value than linear regression when P was limiting. The increase in P∶C with growth was considerably larger in the P fertilization series than in the N fertilization series. As for aboveground data, if the data for excess N and P treatments were included, significant relationships between P∶C and μ disappeared for *Leymus chinensis* and *Cleistogenes squarrosa*.

### Relationships between μ and N∶P of the three species

**Figure 4:**
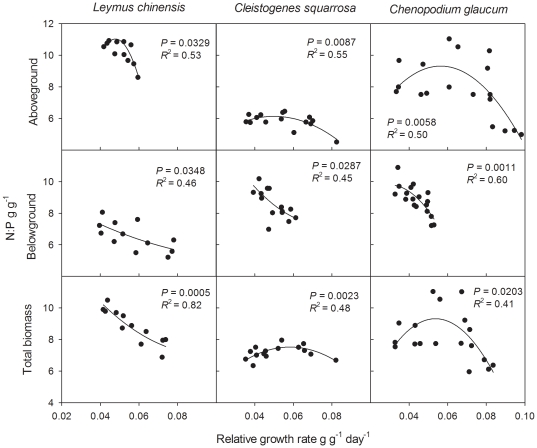
Relationships between relative growth rate and N∶P for aboveground, belowground and total biomass.

Almost all relationships between μ and N∶P of the three species for aboveground, belowground and total biomass were not significant based on equation 3 with the only exception of total biomass of *Leymus chinensis* (*P* = 0.0136, *R* ^2^ = 0.72). However, all relationships of the three species were significant based on equation 4 (Fig. 4). AIC values of quadratic regressions were lower than those of linear regressions. In most cases, the N∶P ratio was a unimodal function of μ, increasing for small values of μ, reaching a maximum, and then decreasing. Although the relationship for all belowground biomass and total biomass of *Leymus chinensis* showed the same trends as predicted by equation 4, we did not find the maximum of N∶P. No significant relationships were found for aboveground biomass of *Leymus chinensis* and *Cleistogenes squarrosa* when data from the excess N and P treatments were included based on equation 4.

### Variation of relative growth rate, N∶C, P∶C and N∶P across the three species

The rank order of aboveground μ for the three species reversed between low and high nutrient conditions (Fig. 1). Growth rate of *Leymus chinensis* was the highest in both the low N and P levels (*P*<0.05, ANOVA; 2 and 0.15 mmol L^−1^, respectively), whereas it was the lowest in high N and P treatments (*P*<0.05, 32 and 4.8 mmol L^−1^ respectively). While the mean value of μ of *Chenopodium glaucum* was the highest in high fertilization treatments for both N and P, it was the lowest in low N and P treatments (Fig. 1). Interestingly, the species rankings for μ for belowground biomass were opposite with that for aboveground.

**Figure 5:**
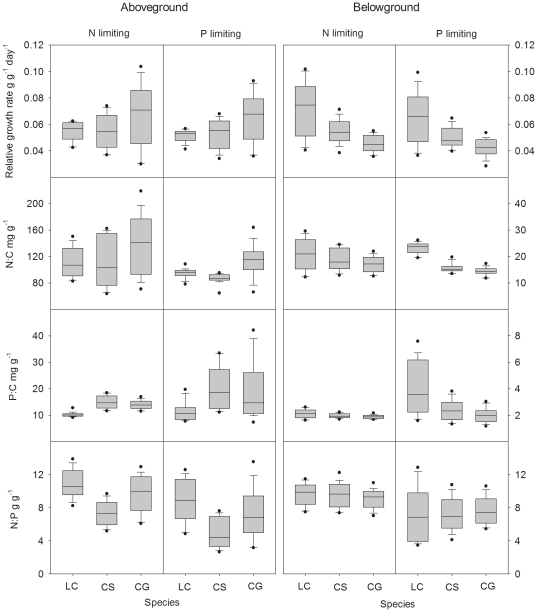
Variation of relative growth rate, N∶C, P∶C and N∶P across the three species.

There were two main trends for the ranges of variation in μ, N∶C, P∶C and N∶P for the three species and aboveground versus belowground. First, the ranges of μ were generally consistent with those of C∶N∶P stoichiometry. Both when N and P were limiting, *Chenopodium glaucum* exhibited the highest range of aboveground μ (Fig. 5), while *Leymus chinensis* showed the lowest range. There were consistent patterns for the three species for ranges of foliar N∶C, P∶C, and N∶P ratios (except P∶C when N was limiting) in both N and P treatments; i.e., *Chenopodium glaucum>Cleistogenes squarrosa>Leymus chinensis* (Fig 5), which was the same as the rank order of ranges of μ. The highest foliar N∶P and the lowest P∶C occurred in *Leymus chinensis*, while the lowest N∶P and the highest P∶C were found in *Cleistogenes squarrosa*. Second, the patterns for belowground biomass were almost completely opposite those just discussed for aboveground biomass (Fig. 5). Overall, belowground biomass of *Leymus chinensis* had the highest ranges of μ, N∶C, P∶C and N∶P, while *Chenopodium glaucum* had the lowest ranges. However, no significant differences were found for N∶P among the three species when N was limiting.

## Discussion

Our results from the sand culture experiment clearly demonstrate strong positive associations of aboveground, belowground and total biomass N∶C and P∶C ratios with μ for these Inner Mongolia grassland vascular plants, which are consistent with numerous previous findings [1], [21], [22], [25], [29]–[31]. Furthermore, when N or P was in excess, positive correlations were lost in *Leymus chinensis* and *Cleistogenes squarrosa*, which is consistent with other research about excess supply of N under strong P limitation [32]–[34], suggesting that excess uptake will confound the physiological need for elements at different relative growth rates with the capacity of excess uptake. For example, if both N∶C and P∶C are taken from P-limited conditions, P∶C describes the physiological need for P but N∶C describes the capacity of N excess uptake. Overall, N∶C ratio was positively related with μ when N was limiting, and P∶C ratio was positively correlated with μ when P was limiting.

As predicted by equation 4, N∶P ratio was a unimodal function of μ, increasing for small values of μ, reaching a maximum, and then decreasing. However, in contrast with Ågren's study, the data did not fit equation 3. Our study suggests that, in vascular plants, N in ribosomes may need to be considered when analyzing the relationship between μ and N∶P [21], at least for grasses in Inner Mongolia grassland. Most studies have shown a negative association between μ and N∶P, not only among plant species but also within populations or cultivars of a given species [1], [10], [27], [29], [33], [34] consistent with the results of belowground and the results of aboveground and total biomass with high μ in our study. Results from this study also provide further support for the suggestion that the negative relationship between μ and N∶P ratio may not hold for plants when P is not limiting [27], likely due to effects of P storage under N limitation. Thus, for vascular plants, μ is positively correlated with N∶P when μ is low while negatively correlated with N∶P when μ is high.

To our knowledge, our data represent the first documentation of the relationships between belowground C∶N∶P stoichiometry and μ, which is consistent with aboveground biomass. In the derivation of equations 1–4, the N∶C is based on the need for proteins for C capture and P∶C is based on the need for ribosomes for protein production [21]. However, roots do not take up C (except for limited uptake of organic compounds). So, the question is why should belowground biomass fit the equations? Likely the answer is that translocation of carbon and nutrients among tissues is a whole-plant process. As μ and C∶N∶P ratios of aboveground biomass were positively related with those of belowground (Fig. 1 and Yu et al unpublished data), belowground biomass should exhibit similar relationships as those seen aboveground.

Across all N treatments, the annual species, *Chenopodium glaucum*, had the highest aboveground μ but had an intermediate N∶P ratio (Fig. 5). *Leymus chinensis* had the highest N∶P while *Cleistogenes squarrosa* had the lowest N∶P but they had similar μ. Similarly, it was difficult to establish clear ranking patterns when we considered N∶C, P∶C and μ both for aboveground and belowground biomass at the inter-specific level. Thus, while data for additional species are clearly needed, across all the nutrient levels, μ was not clearly associated with C∶N∶P ratios at the inter-specific level in our data, consistent with the results from a study focused on 14 *Pinus* species growing at high and low nutrient levels [28]. In contrast, Elser *et al.* [35] found strong and consistent rank order relationships between growth rate, RNA content, and P content (and thus likely N∶P ratio) across five species of *Drosophila*. It is likely that the increased nutrient storage capacities of plants relative to metazoans may underpin this contrast between animal and plant studies.

The reversal in the rank order of aboveground μ of the three species between low and high nutrient treatments (Fig. 1) is notable and suggests an overall performance tradeoff that is manifested when environmental nutrient conditions fluctuate. *Chenopodium glaucum*, an annual species considered to be a fast grower [36], performed well under high nutrient conditions but grew very slowly under low ambient nutrient supply. In contrast, *Leymus chinensis*, a perennial rhizomatous species usually considered as a slow grower [36], grew relatively slowly in highly fertilized conditions but could tolerate poor nutrient conditions. Thus, “fast growers” are not always growing faster than “slow growers” and instead there may be important trade-offs between growth and tolerance that are mediated by above- and below-ground allocation differences (see below). These responses likely reflect inter-specific differences in adaptive strategies, i.e. the “fast growth” species has an advantage in fertile habitats while “slow growth” species are better suited to infertile soils and other stresses related to the efficient husbanding of limiting nutrients [37].

Related to these trade-offs, another notable pattern is that almost all the species ranges for μ, C∶N∶P ratios were opposite between aboveground and belowground biomass, which corresponds well with our previous observations for above- vs below-ground stoichiometric homeostasis (an index of variability in C∶N∶P stoichiometry) [38]. The functional equilibrium model predicts that fertilization will cause reduced allocation to roots [39]–[41] and species that rapidly shift their biomass allocation to aboveground tend to outcompete species with a less responsive root ∶ shoot ratio after N eutrophication [41]. In contrast, slow growers (for aboveground biomass) tend to dominate in arid or low-fertility ecosystems [37]. Our results indicate that these aboveground “slow growers” are likely “fast growers” belowground, a response that may allow them to more effectively exploit below-ground nutrient resources in infertile soils. This corresponds well with the observation that these aboveground “slow growers” have high root ∶ shoot ratios [39], [42].

Consistent with these arguments, the taxon with the highest ranges of μ (*Chenopodium glaucum* for aboveground, *Leymus chinensis* for belowground) tended to also have the highest ranges in N∶C, P∶C and N∶P ratios across treatments, while the species with the lowest ranges of μ tended to have the lowest ranges in C∶N∶P stoichiometry. While more data are clearly needed, these data suggest a pattern in which species-level variation of growth rate is positively correlated with the variation of C∶N∶P stoichiometry. The rank order of stoichiometric homeostasis both for aboveground and belowground biomass of these three species shown in our previous work [38] was the opposite of the rank of variation of relative growth rate in this study, indicating that stoichiometrically homeostasic species are also “homeostatic” in growth rate. Stoichiometric homeostasis in autotrophs, as well as in other organisms, is known to depend on relative growth rate [22], [43], [44]. These studies have shown that, the closer to its maximum capacity a plant grows, the more constrained is its elemental composition. As stoichiometrically homeostatic species tend to be dominant in the community, and homeostatic ecosystems are more productive and stable [45], the ability of species to control their variation of growth rate may be another mechanism responsible for important ecological properties, such as ecosystem structure, functioning and stability.

There are two main limitations to our study. First, because we only studied three species and because newly-germinated perennial plants may not accurately reflect above- and below-ground growth rates and allocation patterns, the relationships between μ and biomass C∶N∶P ratios and the contrasting patterns between above- and below-ground that we document need more testing in future studies. Second, it is preferable to study plant growth as a function of a range of steady-state N and P supplies rather than the pulsed nutrient supply regime in the sand culture approach used in our study [46], [47], because relative growth rates are more closely associated with uptake rates of nutrients rather than with external concentrations supplied under relatively static conditions. If possible, techniques for steady state should be adopted in the future studies testing the growth rate hypothesis for vascular plants.

This study is one of the few evaluations of the GRH in vascular plants and the first to evaluate patterns in belowground biomass. The results showed that, for aboveground, belowground and total biomass, μ was positively correlated with N∶C and P∶C ratios for each of the three species but μ was positively correlated with N∶P ratio in N treatments and negatively in P treatments. No clear associations among μ and C∶N∶P ratios were found at the inter-specific level. Thus, we suggest that the GRH need to be refined for application to vascular plants, likely due to the effects of storage of non-limiting nutrient in plants. More specifically, we propose a revised GRH in which: 1) Plant nutrient contents (N∶C and P∶C) are positively correlated with μ; and 2) There are disproportionate increases in the content of the limiting nutrient relative to the non-limiting nutrient in a cell-quota dependent manner such that the relationship between μ and N∶P ratio is context-dependent.

## Materials and Methods

No specific permits were required for the described field studies and we confirmed that the location is not privately-owned and the field studies did not involve endangered or protected species.

### Sand culture experiment

The sand culture experiment was conducted at the Inner Mongolia Grassland Ecosystem Research Station in 2006. Three plant species, *Leymus chinensis* (a C-3 perennial rhizome grass), *Cleistogenes squarrosa* (a C-4 perennial bunchgrass) and *Chenopodium glaucum* (a C-3 annual species) were selected as our target species, representing the dominant, subdominant, and the annual minor species respectively. Their seeds were planted in pots filled with sand on May 1 and watered with nutrient solutions each day. Sand between 0.2 and 2.0-mm was screened with mesh, and, to minimize the soil nutrient content, washed five times prior to filling plastic pots (30 cm diameter, 35 cm height). For each plant species, we applied treatments consisting of 6 N levels (2, 4, 8, 16, 24, 32 mmol N L^−1^, added as NH_4_NO_3_) and 6 P levels (0.15, 0.3, 0.6, 1.2, 2.4, 4.8 mmol P L^−1^, added as KH_2_PO_4_,) respectively. P concentration was held constant at 1 mmol P L^−1^ in N treatments and N concentration was held constant at 15 mmol N L^−1^ in P treatments. Each experimental pot received the same amounts of macro- and micronutrients except for N and P. The macroelement composition of the solution followed the formula developed by Hoagland and Arnon [48] and the microelement composition was based on Jensen's formula [49]. Each level had three replicates, with three pots randomly allocated to a replicate and a total of 18 pots (3 replicates * 3 pots * 2 harvests) for each species. Each experimental pot received 250-mL of solution every day to maintain a relatively constant macro- and micronutrient concentration (excess solutions were drained through the four holes at the bottom). The pots were washed with 500-ml water twice every 10 days followed by 250-mL solutions immediately to avoid ionic toxicity. All pots were covered when it rained and, if rainwater entered pots, additional 250-mL nutrient solutions were added. There were 10 to 30 individuals in each pot depending on the plant size. The density was controlled to ensure that the plant individuals did not shade each other. The above- and below-ground biomass of thirty plants (within 3 pots) of each plant species for each treatment replicate was harvested on 10 July and 10 August 2006. Healthy, fully expanded leaves and roots were oven-dried at 60°C, powdered and screened with 0.1-mm mesh for chemical analysis (total N and total P).

### Elemental analysis

Total N (% of dry mass) were analyzed with the micro-Kjeldahl method [50] using 2300 Kjeltec Analyzer Unit (FOSS, Sweden). Total P concentrations (% of dry mass) were measured by the ammonium molybdate method after persulfate oxidation [51]. The total C concentration (%) was measured using a modified Mebius method [52]. Briefly, 0.01 g samples were digested with 10 ml 0.50 mol·L^−1^ K_2_Cr_2_O_7_ at 180°C for 5 minutes followed by titration of the digests with standardized FeSO_4_.

### Estimation of relative growth rate

The relative growth rates were calculated by
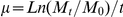
where M_0_ is the initial biomass, M_t_ is the final biomass, and t is the time interval. In this study, M_0_ is the aboveground, belowground or total biomass of each species on 10 July and M_t_ is the biomass on 10 August.

### Statistical analysis

Regression analysis was used to assess the relationships between μ and C∶N∶P ratios (linear regression for μ with N∶C and quadratic regression for μ with P∶C and N∶P). ANOVA was used to test the difference of μ among species. All analysis was performed by SAS (version 9.0, SAS Inst., Cary, NC, USA).
